# Environmental Chlorine Pollution Mitigation Using Material–Pollutant Interactions and Field-Scale Applications

**DOI:** 10.3390/ma19040720

**Published:** 2026-02-13

**Authors:** Ieva Andriulaityte, Marina Valentukeviciene, Ramune Zurauskiene

**Affiliations:** 1 Department of Environmental Protection and Water Engineering, Faculty of Environment Engineering, Vilnius Gediminas Technical University, LT-10233 Vilnius, Lithuania; ieva.andriulaityte@vilniustech.lt; 2 Department of Building Materials and Fire Safety, Faculty of Civil Engineering, Vilnius Gediminas Technical University, LT-10233 Vilnius, Lithuania; ramune.zurauskiene@vilniustech.lt; 3 Department of Landscape Management and Agribusiness Technologies, Faculty of Agrotechnologies, Vilniaus Kolegija, Higher Education Institution, Studentu Str. 39A, LT-14165 Vilnius, Lithuania

**Keywords:** pollutant removal, wasted materials, nature-based solutions, green infrastructure, residual chlorine

## Abstract

Nature-based solutions, including green infrastructure (GI), are considered sustainable tools for stormwater treatment. GI elements (rain gardens, green roofs, etc.) are increasingly applied as integrated approaches for climate change mitigation and environmental pollution reduction. This study focused on investigations of rain gardens for reducing stormwater polluted by residual chlorine after the disinfection of outdoor spaces. Laboratory (column test) and field tests were carried out to evaluate the infiltration capacities of an experimental rain garden model, as well as its efficiency for retaining residual chlorine. The experiments were conducted using simulated rain garden layers composed of waste materials that remained after different production processes. The average infiltration coefficient values obtained were 2.55 × 10^−5^ m/s, 2.45 × 10^−5^ m/s, 2.24 × 10^−5^ m/s, 3.4 × 10^−5^ m/s, 1.28 × 10^−5^ m/s, 1.84 × 10^−5^ m/s (laboratory test), and 1.39 × 10^−5^ m/s (field test). These values correspond to the characteristics of sand–gravel substrates. A chlorine retention efficiency of 82.5–87% was obtained. Granulometric analysis confirmed fraction size suitability for rain garden filtration. This research indicates the potential of rain gardens for reducing stormwater pollution, providing a basis for future research and practical implementation.

## 1. Introduction

Nature-based solutions involving the implementation of green infrastructure technologies are becoming a progressive and innovative solution for reducing water pollution and water scarcity [1,2]. The importance of green infrastructure (GI) in reducing the contamination of surface water bodies is recognized in various European Commission directives [3,4]. GI is considered an advanced method of stormwater management, which, compared with traditional gray infrastructure, offers many additional environmental benefits: it contributes to reducing environmental pollution, mitigating climate change, and increasing the resilience of ecosystems [5,6,7]. At present, GI is gradually being widely accepted and applied in various countries. Scientific research confirms that GI measures are effective and beneficial in reducing stormwater pollution and improving the quality of surface water bodies [8]. This has been proven by various experiments, including laboratory and field tests, and modeling assessments [9,10]. Studies have demonstrated that GI (rain gardens, green roofs, and other vegetated structures) uses natural systems such as vegetation, soil, and permeable surfaces to reduce stormwater pollution and improve the quality of surface water bodies [11,12,13,14,15].

Stormwater treatment is one of the essential elements of urban infrastructure that ensures a safe, healthy, and sustainable living environment. In cities and other urban and residential areas, stormwater flows over impervious surfaces, collecting harmful and toxic pollutants and carrying them into surface water bodies and soil [16]. Today, when most countries are facing the challenge of water scarcity, proper stormwater treatment and reuse are becoming particularly important [17,18,19,20]. Polluted water bodies pose a threat to human health and safety, cause water shortages in various regions, deteriorate water quality, and lead to ecosystem degradation resulting in significant negative environmental and economic consequences. United Nations data indicate that 2.2 billion people still lack access to safely managed drinking water services, while 3.5 billion are without safely managed sanitation services, and the condition of many water basins continues to deteriorate [21]. Therefore, effective stormwater management based on the principles of sustainability and circularity is emerging as an important challenge [22,23]. In June 2025, the European Commission published the European Water Resilience Strategy [24], which calls for action to prevent pollution at water sources and to use advanced technologies and nature-based solutions to reduce pollution in water bodies, and notes that water reuse must be integrated into water resource management. According to European Commission statistics, only 2.4% of wastewater is reused, and this figure varies greatly between member states, ranging from 0 to 80% [25].

Present research investigates stormwater pollution with chlorine-based disinfectants. This form of environmental pollution has not been widely studied but has become more significant during the pandemic, when the intensive use of disinfectants led to increased amounts of residual chlorine in the environment. Various studies have shown that during the COVID-19 pandemic, the use of disinfectants, especially sodium hypochlorite and other chlorine-containing compounds, increased significantly in outdoor and public spaces. Disinfectants containing chlorine compounds are also used in industry and to ensure the safety of swimming pools and other recreational water systems. When these harmful substances transfer to pavements, streets, or other impervious surfaces, they are easily washed by runoff during rainfall and enter surface water bodies. Since both direct and indirect runoff are discharged into rivers and lakes, aquatic ecosystems are at risk of being contaminated with chemical disinfectants [26,27]. Studies show that chlorine-based disinfectants pose a threat to aquatic fauna and flora, pose potential risks to urban wildlife, and can affect food and water sources [28,29,30]. When chlorine enters water, it combines with other substances that are present to form harmful compounds, such as trihalomethanes, chloramines, etc., which are harmful to aquatic ecosystems [31,32]. Chlorine also reacts with organic matter in stormwater, forming organic chlorine compounds that remain in the environment as pollutants and can pose a significant threat to aquatic ecosystems [33]. Slaterry et al. note that the formation of THMs and HAAs increased with increasing chlorine dose, e.g., at 3 mg/L chlorine, 20 μg/L THMs were formed, compared with 10 μg/L at 0.5 mg/L chlorine (THMs (trihalomethanes) and HAAs (halogenated acetic acid compounds). It was found that chlorine reacts with organic matter in water, forming roughly 800 disinfection by-products [34,35]. Disinfectants can also corrode the surface of different materials. Studies show that further research is needed to assess the impact of disinfectants on surfaces that are frequently and continuously exposed, as these substances may still have negative effects [36]. Thus, the increased use of disinfectants during the pandemic highlighted their link to surface water body pollution and further reinforced the need to develop alternative, sustainable solutions for chlorine removal from stormwater. Previous studies have analyzed the following aspects: the interaction of residual chlorine with chemical elements in stormwater after surface disinfection [37]; potential filtration materials and their ability to remove chlorine from stormwater [38]; the application of bentonite clay for chlorine removal from stormwater [39]; the interaction of chlorine and microelements in plants; and the ability of plants to retain and accumulate chlorine [40]. Considering the results of former experiments, the present research aims to assess the efficiency of a rain garden as an element of green infrastructure in retaining residual chlorine formed after surface disinfection with sodium hypochlorite solution.

The novelty of the present work is based on research into and tests on chlorine removal from stormwater using green infrastructure technology—an experimental rain garden model. It allows us to assess how material solutions, such as the use of waste materials, can increase the efficiency of pollution reduction and ensure sustainable solutions. This is important because, first, stormwater enters water bodies that could be used for drinking and recreational purposes. Secondly, even low concentrations of chlorine in stormwater cause ecological stress; it oxidizes organic matter and forms toxic disinfection by-products. Thirdly, chlorine compounds are highly reactive, so even temporary entry into water bodies can disrupt microbiological processes. Several studies have suggested that rain gardens might reduce stormwater pollution by up to 80–90% [41]. Materials should be selected according to their particle size distribution and resistance to pollutant retention [42,43]; therefore, it is necessary to assess materials’ efficiency for retaining pollutants. Research recommends using local, recyclable filtering materials for rain garden construction layers [44]. A rain garden collects and filters stormwater before it enters water bodies. It is not only a technical solution for stormwater management, but also a tool for sustainable urban planning, helping to restore the ecological balance that has been disrupted by intensive urbanization and climate change [45]. The rain garden is an innovative and sustainable means of stormwater management; therefore, its study allows us to assess how material solutions, such as the use of waste materials, can increase the efficiency of pollution reduction and contribute to the creation of green infrastructure as well as the achievement of sustainability goals. However, there is still a lack of scientific data on the use of various materials, soils, and other components for rain garden construction. This remains one of the main challenges limiting the application of effective approaches and the implementation of European Green Deal requirements.

## 2. Materials and Methods

Laboratory and field experiments were carried out to assess the infiltration capacities of the rain garden model and its efficiency for retaining residual chlorine.

### 2.1. Laboratory Test: Determination of Infiltration Coefficient and Residual Chlorine Retention

**Figure 1:**
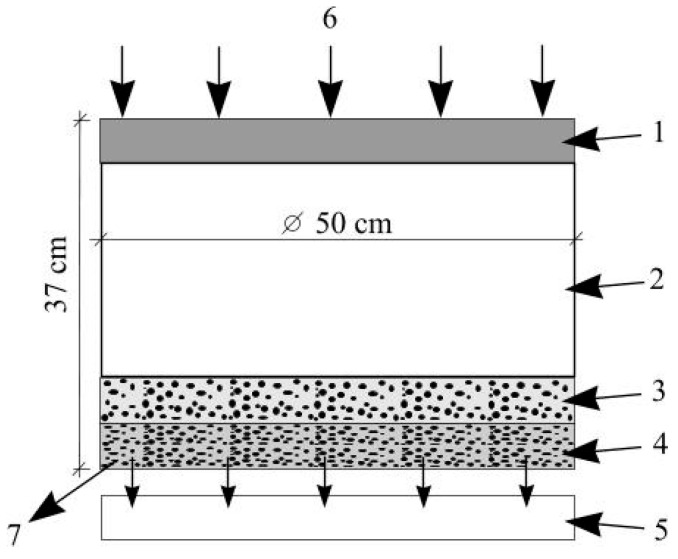
Column test: 1—mulch layer; 2—filtration layer; 3—supporting layer; 4—drainage layer; 5—sampling tank; 6—collected stormwater; 7—filtered water.

Following other studies [46,47,48] to determine the infiltration coefficient, a vertical column was installed in the laboratory. To simulate a typical rain garden layer structure, the column was filled with four layers: a drainage layer (expanded clay aggregate, 4–10 mm, 5 cm) designed to provide structural strength to the substrate and prevent fine particles from sliding into the drainage area; a filtration layer (substrate, mixture of fine gravel and sand, 0.1–8 mm, 22 cm); and a mulch layer (pine sawdust, fraction size 0.1–20 mm, 5 cm) (Figure 1). The inner diameter of the experimental column was 50 cm, and the total height was 37 cm. The aim of the laboratory test was to determine the effectiveness of the rain garden’s filtration layer and whether the system can be used for residual chlorine removal. Infiltration capacities were tested using collected stormwater; pH and conductivity (μS/cm) were measured before and after the test. Measurements were collected using a portable conductivity measurement device (Cond315i, WTW, Weilheim, Germany) and a pH meter instrument (WTW pH 323, WTW, Weilheim, Germany). In order to ensure the reliability of measurements, the stormwater indicators (pH; conductivity, μS/cm; COD, mg/L; BOD_7_, mg/L; odor, score) were determined in an accredited laboratory in accordance with international standards (EN ISO/IEC 17025) [49]

The efficiency of residual chlorine retention was determined using the same column. The test was performed as follows: 1. Collected stormwater was prepared and water parameters (pH; conductivity, μS/cm) were measured (control test). 2. Collected stormwater was contaminated with residual chlorine (10 mg/L) and water parameters were measured (pH; conductivity, μS/cm; residual chlorine, mg/L). 3. Collected stormwater was poured into a column and filtered, and water parameters (pH; conductivity, μS/cm; residual chlorine, mg/L) were measured. Residual chlorine concentrations were measured using a chlorine meter (CL200 ExStik, measuring range of 0.01 ppm–10 ppm, Extech, Beijing, China). Based on the obtained residual chlorine values before and after filtration, the residual chlorine retention efficiency was evaluated. The assessment was carried out using collected stormwater polluted by sodium hypochlorite. Laboratory experiments were conducted using approximate concentrations of 10 mg/L. This corresponds to the real levels of residual chlorine that can enter from stormwater during surface disinfection and from swimming pools. The aim was to determine the capacities of simulating rain garden layers to retain residual chlorine. The infiltration coefficient was calculated according to the following formula [50]:

$$U=-\;K\frac{dh}{dl},m/s$$

where *K*—hydraulic conductivity; *dh*/*dl*—hydraulic gradient.

Materials used in experiments: Substrate (mixture of sand and gravel, fraction—0.1–8 mm) consists of a fine fraction and a coarse fraction. Fine fraction—0.1–2 mm—consists of sand containing feldspars, quartz, magnesite, granite, and other rock particles.

Coarse fraction is 2–8 mm in size. These are rocks that have disintegrated by natural erosion, the surfaces of which are polished and sufficiently smooth. The number of broken particles is up to 10%. The bulk density of this mixture is 1750 kg/m^3^ and the density is 2650 kg/m^3^.

Expanded clay (0.1–4 mm) particles have a round shape. The surfaces of the particles are sintered, with a small number of open pores. The main oxides of expanded clay are as follows: SiO_2_—55%, Al_2_O_3_—28,% Fe_2_O_3_—4%, Fe_3_O_4_—3%, Na_2_O—3%, K_2_O—2,% MgO—2%, others—about 3%. The expanded clay mixture consists of fine-fraction particles 0.1–2 mm and coarse particles 2–4 mm. Expanded clay density: 550 kg/m^3^. Compressive strength: 2 MPa. Gravel consists of particles with a size of 8–11 mm.

Gravel particles are round, polished, and made of sedimentary rocks. Gravel contains the remains of quartz, limestone, magnesite, and granite particles, which, during decomposition and weathering, were formed from larger stones and formed into layers carried by water, which is why the surfaces of these particles are polished and smooth, with small depressions. The bulk density of gravel is 1520 kg/m^3^ and the density is 2610 kg/m^3^.

Sawdust is obtained from pine wood. Particle size 0.1–20 mm. Density of pine wood: 470 kg/m^3^. Density of sawdust: 80 kg/m^3^. Particle thickness: 0.1–2 mm. Particle length: up to 20 mm.

Microscopic assessment of materials was conducted using Digital Microscope RoHS, Beijing, China. The software used was Setap Software S4 (HiVew20230724).

### 2.2. Rain Garden Experimental Model

The experiment’s location coordinates were 54.718421, 25.18648 (Figure 1), Buivydiskes, Vilnius district, Lithuania. Experiment time: April–November, 2025. Experiment time was selected following previous experiments [51,52].

The experimental rain garden model (1 × 3 m) was installed in accordance with the design recommendations presented in the scientific literature [53]. We used an intermediate, semi-controlled rain garden model designed to evaluate infiltration processes and residual chlorine behavior under more realistic and controlled conditions. This study design allowed for a reduction in the impact of external factors and a more accurate determination of the influence of filtration layers and hydraulic conditions on the studied processes.

**Figure 2:**
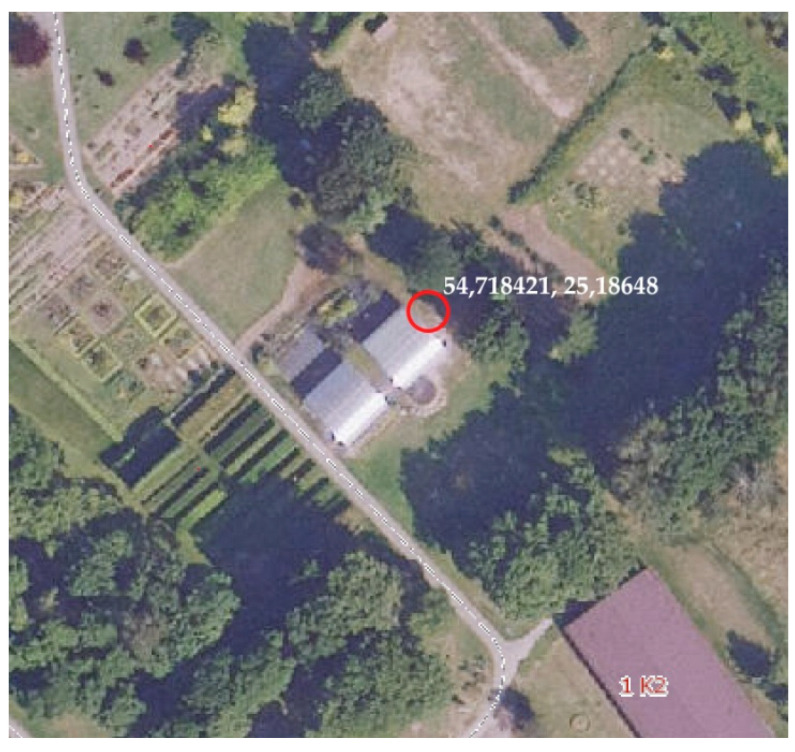
Experiment location.

The test site was selected near impermeable surfaces and a stormwater collection area. A rectangular rain garden shape (3 m^2^) was selected for the experimental model, with a total depth of 1 m. Later, it was enlarged to (10 m^2^). A gutter was installed for water inflow, connecting the downpipe with the garden, and an outflow zone was formed such that excess water could drain in a controlled manner without damaging the surrounding areas. In the case of the experimental model, a 2% slope was applied and the slopes were reinforced with stones to prevent soil sliding. The rain garden was installed according to the following stages: the contour was marked, the top layer of soil was removed, a depression was formed, the inflow and outflow zones were installed, the garden layers were formed, and local plants were planted. The main rain garden layers are shown in Figure 2 [54].

**Figure 3:**
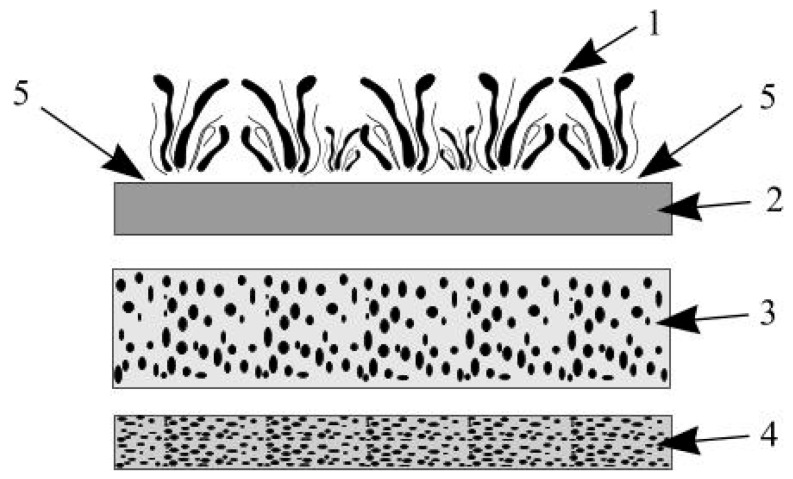
The main rain garden layers: 1—plants; 2—mulch; 3—filtration layer; 4—drainage layer; 5—stormwater.

The following filtration layers were used to construct the experimental rain garden (Figure 3): a drainage layer (10 cm of expanded clay granules); a filtration layer (20 cm of bentonite); a soil substrate (60 cm); seven species of native plants; and a mulch layer (10 cm of pine sawdust) to protect against erosion, moisture evaporation, and weed growth. The substrate’s particle size was measured using granulometric analysis (sieve test), which ensured that the selected soil substrate had the appropriate hydraulic conductivity and structural stability necessary for effective infiltration and contaminant retention. Previous studies have stated the importance of substrate granulometric analysis because the particle size distribution influences the pore network, which determines the water infiltration rate and hydraulic conductivity [55].

Infiltration capacities were determined using the experimental rain garden model. Stormwater was collected in a special 40 L container. The test water was poured into the rain garden. The time it took for the water to absorb was measured, changes in the water level were recorded at time intervals, and the infiltration coefficient was calculated according to the formula presented in [50].

## 3. Results and Discussion

### 3.1. Laboratory Test

#### 3.1.1. Determination of Infiltration Coefficient

Distilled water (10 L) was used to determine the infiltration capacities of the rain garden layers. Nine measurements were collected and the maximum, minimum, and average Q (L/s) values were determined. The total duration of the experiment was about 1.5 h (Table 1, Table 2 and Table 3).

The obtained data shows that the average flow rate in the first experiment was 0.005 L/s; in the second—0.0048 L/s; and in the third—0.0044 L/s. The first experiment showed the highest infiltration rate, while the slowest infiltration rate was obtained in the third experiment. The average infiltration values show that the substrate is sufficiently permeable. The average infiltration coefficient values (2.55 × 10^−5^ m/s; 2.45 × 10^−5^ m/s; and 2.24 × 10^−5^ m/s) matched the hydraulic properties of sand–gravel. Studies have confirmed that sand increases infiltration [56]. The results show that the selected layers are suitable for a rain garden and have adequate hydraulic permeability. From repeated tests, the average infiltration rate, 0.00667 L/s, and infiltration coefficient, 3.4 × 10^−5^ m/s, were determined. After the repeated addition of 10 L of water, the infiltration rate decreased to 0.0025 L/s and the infiltration coefficient value to 1.28 × 10^−5^ m/s. Comparing both tests’ values, the infiltration coefficient decreased by approximately 62%. In the first test, the substrate was drier, which resulted in higher infiltration, while in the second test, the infiltration decreased due to the substrate’s pore saturation. This simulates real natural conditions, when stormwater after a dry period infiltrates faster and then slows down. The infiltration coefficient values of 3.4 × 10^−5^ m/s and 1.28 × 10^−5^ m/s correspond to values typical for rain garden substrates [49]. The filtration layer had a high initial water absorption rate, which decreased as the substrate became saturated, the obtained infiltration rate was 0.0036 L/s, and the infiltration coefficient was 1.84 × 10^−5^ m/s. This is consistent with previous experiments’ results and confirms that the analyzed layers can be applied in a rain garden system [37,40].

#### 3.1.2. Determination of Residual Chlorine Retention

The results for residual chlorine retention (different concentrations) are presented in Table 4 and Table 5.

The results confirm previous experiments [37] presenting the changes in stormwater indicators after water contamination by sodium hypochlorite. Table 4 indicates that indicators of the control water and the contaminated water changed slightly: pH decreased from 7.28 to 6.68 and, after filtration, the pH value was 5.68. The explanation for this may be that the rain garden layers retained residual chlorine and the change was caused by the substance’s reaction to chlorine. Comparing the control water and contaminated water also shows a fixed increase in conductivity due to dissolved ions. The highest conductivity value was obtained after filtration (337 μS/cm); this increase in conductivity can be explained by changes in the chemical structure of the filter material. Sodium hypochlorite decomposes into ionic forms, potentially releasing soluble salts and ions from the rain garden layers. Table 4 presents the 87% efficiency of the rain garden layers to retain residual chlorine. Experiments with collected stormwater samples showed similar results. Stormwater indicators were analyzed in a certified laboratory (Table 5).

The results of the tests show that the control sample was characterized by an almost neutral pH (7.3), an average electrical conductivity (287 µS/cm), and an absence of odor, which indicates good initial water quality. In the sample contaminated with sodium hypochlorite, increased chemical oxygen demand (COD—46 mg/L) resulted in a noticeable chlorine odor (1 point). After filtration, the pH remained stable (7.7) while the conductivity increased to 426 µS/cm. This increase can be explained by the release of dissolved ions from the filter materials. Biochemical oxygen demand (BOD_7_—13 mg/L) also increased. Table 5 presents an 82.5% efficiency for residual chlorine retention. The experimental results show that the filtration system was effective in reducing the residual chlorine concentration and significantly affected the main stormwater indicators, stabilizing pH and changing the chemical composition of water.

Residual chlorine retention efficiency was determined to be 82.5–87%. Other studies have confirmed the efficiency of rain gardens for reducing stormwater pollution [41,61].

#### 3.1.3. Microscopic Analysis of Materials

**Figure 4:**
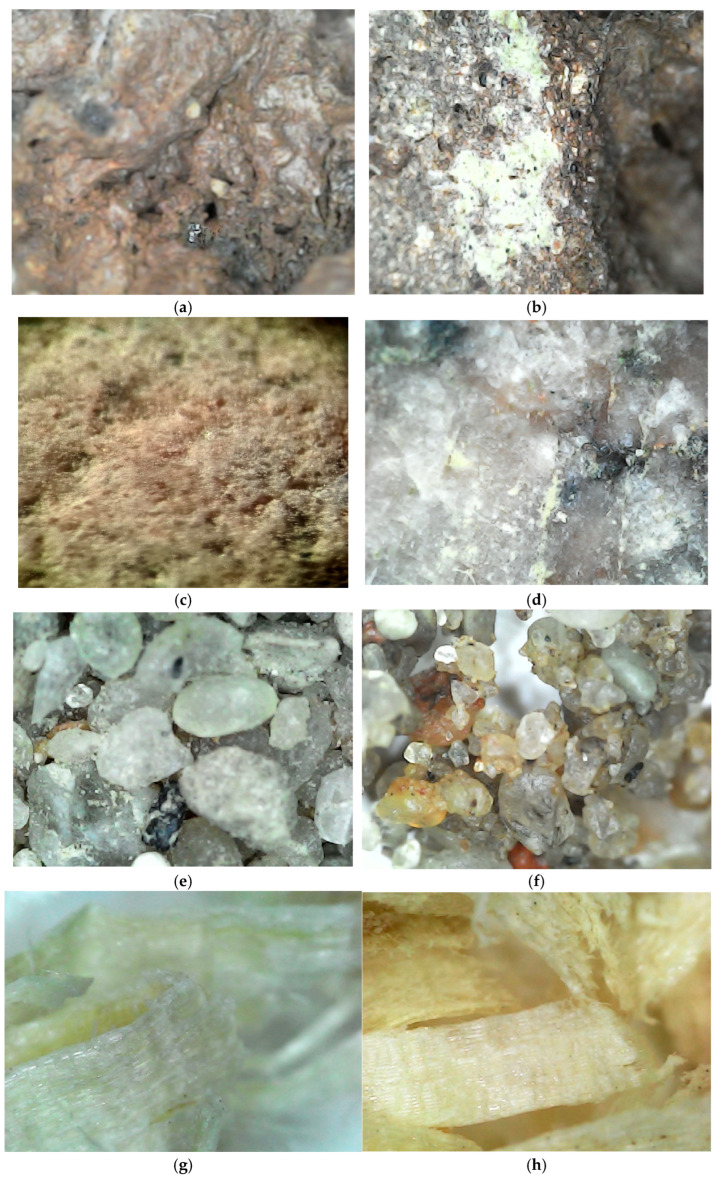
Microstructure analysis of the surface structures of the rain garden layer materials before and after interacting with sodium hypochlorite solution: (**a**) expanded clay before interaction with residual chlorine, (**b**) expanded clay after interaction with residual chlorine; (**c**) gravel; before interaction with residual chlorine, (**d**) gravel after interactin with residual chlorine (**e**) substrate; before interaction with residual chlorine, (**f**) substrate after interaction with residual chlorine (**g**) sawdust before interaction with chlorine, (**h**) sawdust after interaction with residual chlorine.

The microscopic analysis of the rain garden layer materials before and after interaction with chlorine showed structural changes (Figure 4).

Figure 4a shows that the surface of the expanded clay before interaction with residual chlorine was more uniform, the structure was more compact, and there were fewer fresh inclusions and deposits. After reaction with chlorine (Figure 4b), the surface becomes lighter and pores and microcracks become visible. Expanded clay particles exposed to chlorine compounds are covered with a thin shiny film, while expanded clay particles have a sintered brown surface, and inside the particles there is a black porous structure. Expanded clay particles with an unbroken surface structure have few open pores and voids; if the particle surface is cracked, then a larger number of internal voids are exposed, which are connected. These changes are associated with the mineral composition of expanded clay, which is dominated by SiO_2_ and Al_2_O_3_, and the presence of Fe oxides, which can form active surface sites for catalytic processes. Such surface changes indicate not only mechanical deposition, but also a possible chemical interaction with residual chlorine, which contributes to its retention [62].

Gravel particles (Figure 4c) have a very different external surface depending on the origin of the particle. Some particles have a porous surface, which are particles of carbonate sedimentary rock origin, while some particles have a smooth surface, which are particles of magmatic origin. Because of this, the effect of chlorine-containing liquid on them is different. Gravel particles without chlorine are more uniform and even, and less sediment is visible. After exposure to chlorine (Figure 4d), translucent plaque appeared in the depressions of particles with a smooth surface, which accumulated and crystallized. The particles with a porous surface did not change; only a thin shiny surface layer appeared on them and small microstructural changes were visible. Although microstructural changes were limited, these observations, together with the mineral composition (quartz, limestone, and magnesite) [63], indicate that gravel participates in chlorine retention processes through surface sorption and sediment formation.

Optical microscopy analysis of the substrate shows that the substrate surface is structurally stable and unchanged (Figure 4e). Sand and gravel grains are less interconnected and clear gaps are visible. After contact with chlorine (Figure 4f), signs of surface deposits and aggregation of fine particles appeared on the surface of the sand–gravel substrate. Compared with the initial sample, the structure became more heterogeneous, but no degradation of the mineral framework was detected. This allows us to assume that sorption and oxidative processes contribute to the retention of residual chlorine.

Pine sawdust analysis (Figure 4g,h) showed that before the interaction with chlorine, the structure of the sawdust was uniform and layered, the fibers were elastic, with a smoother surface, the color was light, whitish, without bright spots, and the surface appeared less damaged, with a clearly recognizable fibrous texture. After interaction with chlorine, the structure of the sawdust becomes rougher, is less uniform, microcracks and irregularities are visible, the color is more yellowish and brownish in places, and the surface is more porous. These changes are associated with the oxidation of organic matter and functional groups (e.g., hydroxyl and carboxyl), which create favorable conditions for the chemical binding of residual chlorine. This allows us to assume that sawdust acts not only as a physical filtration component but also as a reactive medium contributing to the reduction in residual chlorine.

The optical microscopy results confirm the formation of surface changes and deposits in the filter media. Sorption and oxidation processes are considered to be the dominant mechanisms for the retention of residual chlorine, and the results obtained are consistent with advanced solutions for the removal of chlorine and other contaminants described in the literature [64,65]. Future studies are planned to apply more detailed and advanced methods (e.g., SEM–EDS, XRD, and FTIR) in order to substantiate the proposed mechanisms in more detail.

### 3.2. Field Test

#### Determination of Infiltration Coefficient

Stormwater (40 L) was used to determine the infiltration coefficient. Test samples were collected in the rain garden location at stormwater outlets. Table 6 presents stormwater indicators before and after filtration. The results show that some stormwater indicators (pH, turbidity, and color) did not change significantly. pH increased slightly (before filtration 6.8, after—7.0), which indicates that the filtration materials are not acidic and are chemically stable. Meanwhile, other indicators (conductivity, COD) changed significantly. The increase in conductivity might be associated with the leaching of dissolved ions from the substrate. A slight increase in biochemical oxygen demand confirms the presence of biodegradable organic substances.

Stormwater was poured into the rain garden. After 5 min from the first water pouring, the first filtrate was recorded at the water collection tank. Later, water was poured in stages: 10 L was poured, and then an additional 10 L, and another 10 L (a total of 30 L). After 11 min from the start of the test, the filtered water began to be collected into a container for continuous monitoring. Later, another 10 L was additionally poured in small portions; it was observed that the water level in the column gradually rose. Within 16 min from the start of the test, a total of 40 L of water was poured into the column. After 17 min, a ~5 cm layer of water was recorded above the surface of the filtration medium. After 40 min from the start of the test, 1 L of water was taken/removed from the system, and the remaining water layer was about ~2.5 cm; this level did not increase any more, which indicates a stabilized state under the applied inlet regime. The infiltration rate was 0.047 L/s and the infiltration coefficient was 1.39 × 10^−5^ m/s. The obtained value is close to the values of sand–gravel substrates. The infiltration coefficient indicates that the substrate is suitable for rain garden system. Residual chlorine retention was approximately 90%.

Laboratory and field experiments were conducted to evaluate the infiltration properties of the rain garden model and its efficiency in retaining residual chlorine. In this study, the laboratory column was designed to understand the mechanisms of the process under controlled conditions, while the field rain garden model was designed to evaluate more realistic conditions. The results will allow us to describe the convergence of trends—in particular, the reduction in residual chlorine and changes in certain water quality parameters after filtration

**Figure 5:**
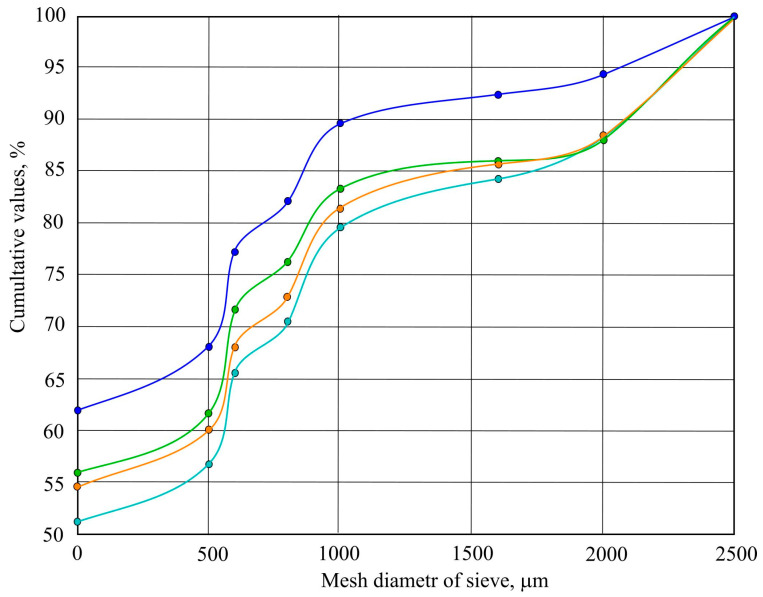
Distribution curve of substrate grain size: blue color—sample 4; orange color—sample 2; green color—sample 3, dark blue color—sample 1.

Granulometric analysis (Figure 5) is an important element in assessing the structure of a material, which affects the infiltration rate and the efficiency of contaminant retention. It allows us to assess whether a fine particle will clog the drainage layer and fill the pores, which is important for ensuring the effective operation of the rain garden. Particle size affects the aeration of plant roots, oxygen access, and the availability of water and nutrients.

The granulometric analysis of the substrate showed that most of the substrate consists of small particles, smaller than 0.5 mm. It was determined that about 88–94% of the mass passes through a 2 mm sieve, 80–90% through a 1 mm sieve, and 51–62% through a 0.5 mm sieve. The higher curve of the graph shows that the fractions have a higher cumulative mass, especially in finer sieves (0–800 µm). The other curves show that the values are close to each other, which indicates good homogenization of the material and repeatable granulometric composition. The substrate is sandy and homogeneous, and there are few gravel particles, which indicates good water permeability. It is concluded that the substrate is suitable for use as a filtration layer in a rain garden. This ensures rapid infiltration and reduces the risk of clogging, and the fine fraction has a good level of residual chlorine retention.

The obtained results will be useful in the future investigation and installation of rain gardens to ensure the development of nature-based solutions for sustainable stormwater treatment.

## 4. Conclusions

Laboratory test results indicated that the analyzed rain garden layers might be characterized by sufficient and stable hydraulic permeability. The average values of the infiltration rate (0.005 L/s; 0.0048 L/s; 0.0044 L/s) and infiltration coefficient (2.55 × 10^−5^ m/s; 2.45 × 10^−5^ m/s; 2.24 × 10^−5^ m/s) correspond to the hydraulic properties characteristic of sand–gravel substrates. It can be concluded that the selected filtration medium is suitable for rain garden systems. The experiments showed that a drier substrate ensures a higher initial infiltration rate and, as the pores are saturated with water, the infiltration gradually decreases. This dynamic corresponds to real natural conditions and shows that the system is able to adapt to a changing precipitation regime. Repeated tests and experiments with stormwater samples confirmed the repeatability and stability of the results (3.4 × 10^−5^ m/s; 1.28 × 10^−5^ m/s; 1.84 × 10^−5^ m/s).

The infiltration rate (0.047 L/s) and infiltration coefficient (1.39 × 10^−5^ m/s) determined in field tests are close to the values typical for sand and gravel substrates and confirm that the selected substrate composition is suitable for rain garden systems. The obtained results show that the water does not stay in the system for too long, but sufficient contact time is maintained, creating conditions for effective pollutant retention. Therefore, the studied rain garden layer model is hydraulically suitable and can be successfully applied for rainwater management and treatment.

Laboratory and field tests confirmed that the rain garden layer system ensures adequate infiltration and is effective in reducing residual chlorine concentration. The residual chlorine reduction efficiency was approximately 80–90%.

Granulometric composition analysis showed that the substrate has good water permeability and is suitable for use as a filtration layer in rain gardens.

Future research is required to apply these outcomes to the selection of other materials capable of effectively removing residual chlorine and other stormwater micropollutants.

## Figures and Tables

**Table 1:** Primary experimental results for infiltration rate (runs I, II, and III).

Parameters	I	II	III
Time (min and s)	8.35	8.50	4.44
Q (L/s)	0.0075	0.00625	0.0015
Collected test water (mL)	3900	3000	440

Average infiltration coefficient, m/s—2.55 × 10^−5^.

**Table 2:** Secondary experimental results for infiltration rate (runs I, II, and III).

Parameters	I	II	III
Time (min and s)	9.42	8.30	11.40
Q (L/s)	0.00552	0.0049	0.004
Collected test water (mL)	3200	2500	2700

Average infiltration coefficient, m/s—2.45 × 10^−5^.

**Table 3:** Tertiary experimental results for infiltration rate (runs I, II, and III).

Parameters	I	II	III
Time (min and s)	11.33	9.20	7.46
Q (L/s)	0.0048	0.0046	0.0038
Collected test water (mL)	3300	2500	1740

Average infiltration coefficient, m/s—2.24 × 10^−5^.

**Table 4:** Primary experimental stormwater indicators.

Stormwater Indicators	Control Sample	Contaminated Test Water Before Filtration	Contaminated Test Water After Filtration
pH	7.28	6.68	5.68
Conductivity, μS/cm	2.9	17.8	337
Residual chlorine, mg/L	-	3.17	0.41

COD below limit of confidence.

**Table 5:** Secondary experimental stormwater indicators.

Stormwater Indicators	Control Sample	Contaminated Test Water Before Filtration	Contaminated Water Test After Filtration	Research Methodology
pH	7.3	7.8	7.7	[57]
Conductivity, μS/cm	287	301	426	[58]
COD, mg/L	<30	46	52	[59]
BOD_7_, mg/L	7.8	5.2	13	[60]
Odor, score	Odorless	Chlorine 1	Odorless 0	
Residual chlorine, mg/L	-	1.83	0.32	

**Table 6:** Tertiary experimental stormwater indicators.

Stormwater Indicators	Control Sample	Test Water After Filtration	Research Methodology
pH	6.8	7.0	[57]
Conductivity, μS/cm	53	284	[58]
COD mg/L	<30	46	[59]
BOD_7_	3.8	4.6	[60]
Residual chlorine, mg/L	10.0	1.0	

## Data Availability

The original contributions presented in this study are included in the article. Further inquiries can be directed to the corresponding author.
